# Transradial approach in vertebral artery stenting

**DOI:** 10.3389/fneur.2025.1643105

**Published:** 2025-09-12

**Authors:** Yao Zhang, Wenya Lan, Hui Liu, Kang Yuan, Ruidong Ye

**Affiliations:** ^1^Department of Neurology, Pukou Hospital of Chinese Medicine Affiliated to China Pharmaceutical University, Nanjing, China; ^2^Cerebrovascular Disease Center, Nanjing Brain Hospital Affiliated to Nanjing Medical University, Nanjing, China; ^3^Department of Neurology, Affiliated Changshu Hospital of Nantong University, Suzhou, China; ^4^Department of Neurology, Jinling Hospital, Affiliated Hospital of Medical School, Nanjing University, Nanjing, China

**Keywords:** vertebral artery, stent, radial, stroke, endovascular treatment

## Abstract

**Background:**

The transradial approach (TRA) has been gaining attraction for endovascular procedures. This study aimed to compare the efficacy and safety of the TRA vs. the transfemoral approach (TFA) for stenting in patients with vertebral artery (VA) stenosis.

**Methods:**

We retrospectively enrolled consecutive patients treated with endovascular stenting in the V1 segment of the vertebral artery from August 2020 to October 2021. We divided patients into two groups (TRA group and TFA group) and compared the procedure outcomes, post-procedure complications, and long-term outcomes with logistic regression models and propensity score-matching methods.

**Results:**

Among the 109 enrolled patients, 45 patients were treated via TRA and 64 patients were treated via TFA. The TRA group had a significantly shorter procedure time (43 vs. 50 min, *p* = 0.005) and a shorter length of stay than the TFA group. The post-procedure complications were slightly higher in the TFA group than in the TRA group (10.9% vs. 2.2%, *p* = 0.179).

**Conclusion:**

This study found that TRA was a safe and feasible approach for vertebral artery stenting of V1 segment stenosis. TRA demonstrated advantages in reducing procedural time and length of stay compared to TFA. However, further large-scale and randomized controlled studies are warranted to verify these findings, given the retrospective and non-randomized nature of this study.

## Introduction

Approximately 20% of ischemic stroke subtypes occur in the posterior circulation, and vertebral artery (VA) stenosis is a significant but often underestimated cause of posterior ischemic strokes (1, 2). The V1 segment of the vertebral artery originates from the ostium of the fifth or sixth cervical vertebrae. Due to the curved anatomy and slow blood flow velocity of the V1 segment,it is the most frequent site of severe occlusive lesions in the vertebral artery (3) and is associated with an increased risk of poor functional outcomes and mortality compared to carotid circulation infarctions (4). However, the optimal treatment for vertebral artery stenosis remains controversial. On one hand, open surgery for V1 stenosis is constrained by the incidence of perioperative complications (5); on the other hand, endovascular treatment with stenting is an attractive treatment option because of its safety and higher rates of technical success (6, 7). However, the efficacy of stenting remains uncertain due to the complex vascular anatomy and the risk of in-stent restenosis (ISR) (8, 9).

The transradial approach (TRA) has been gaining attention among interventional physicians in recent years as an alternative artery access for endovascular treatments in recent years (10). Neurointerventional physicians have explored the feasibility of TRA in various procedures, including diagnostic cerebral angiography, aneurysm clotting, and endovascular stenting (11–14). Previous studies have reported several advantages of TRA over the transfemoral approach (TFA) in terms of direct access to the vertebral artery, easy navigation to the complex arch anatomy, and fewer perioperative complications (15). However, prospective and randomized evidence remains lacking, and few studies have investigated the feasibility of endovascular treatment via TRA in V1 segment stenosis. Hence, we conducted a retrospective and non-randomized study, reporting our multicenter experience in comparing the efficacy and safety of TRA vs. TFA for stenting in patients with V1 segment stenosis.

## Methods

### Study population

We enrolled patients treated with endovascular stenting in the V1 segment of the VA from a prospective registry conducted at three comprehensive centers (Nanjing Brain Hospital, Affiliated Changshu Hospital of Nantong University, and Jinling Hospital) between August 2020 and October 2021. This study was approved by the institutional ethics review board of the Nanjing Brain Hospital and other participating centers. All patients signed written informed consent forms before undergoing surgery. This study was conducted in accordance with the 1964 Helsinki Declaration and its later amendments, or with comparable ethical standards.

Patients were required to meet the following criteria: (1) aged ≥18 years; (2) diagnosed with ischemic stroke ≥8 days after onset; (3) underwent brain plain scans and angiographic examinations before or after admission; (4) diagnosed with symptomatic stenosis (lesions of 50–99%) or asymptomatic stenosis (lesions ≥70%) in the V1 segment of the vertebral artery. We excluded patients according to the following criteria: (1) experienced a new major stroke within 3 months before onset; (2) had lesions related to non-atherosclerotic diseases, such as arteritis; (3) had the presence of aneurysms, cerebral arteriovenous malformations, or intracranial tumors; (4) had incomplete angiographic and clinical follow-up information.

### Vertebral artery stenting

The procedures were performed by experienced neurointerventionalists, each of whom had completed at least 50 angiographic or stenting procedures via both TRA and TFA. We reviewed each participating surgeon’s experience, categorizing it into three tiers: 50–100, 100–150, and >150 procedures. Prior to the procedure, patients received dual antiplatelet therapy consisting of aspirin (100 mg/day) and clopidogrel (75 mg/day) for at least 5 days, with cilostazol or ticagrelor used as alternatives based on platelet function testing.

The procedures were routinely performed under local anesthesia at the puncture site: distal or proximal radial artery, or the femoral artery. Systemic heparinization was administered to maintain the activated coagulation time between 250 and 300 s. TFA was performed according to standard guidelines via the common femoral artery. For TRA, operators performed a modified Allen test or reverse Barbeau test to evaluate the collateral circulation in the hand before the procedure. After administering local anesthesia, the radial artery was punctured using the modified Seldinger technique. Nitroglycerin was infused through the introducer sheath (Radifocus Introducer II, Terumo, Tokyo, Japan) to prevent radial vasospasm.

During the procedure, the operators placed a 6F guiding catheter (Cordis, Miami Lakes, FL, USA) into the subclavian artery or before the ostium of the VA. They used a 0.014-inch guidewire to pass through the lesion under roadmap guidance. The choice of angioplasty balloon sizes for pre-dilation and post-dilation, as well as the stenting devices [e.g., the Express Vascular SD (Natick, MA, USA), the Biotronik (Woermannkehre, Berlin, Germany)], was based on clinical experiences and lesion morphology. After the procedure, hemostasis of the puncture site was achieved through manual compression or compression devices. Access site complications, such as radial artery occlusion, were evaluated using Duplex ultrasonography or the Reverse Barbeau test, as per center preference. Duplex ultrasonography offered structural visualization of the radial artery and assessed blood flow using color and pulsed Doppler imaging, with the absence of flow confirming occlusion. The Reverse Barbeau Test, a convenient method with lower accuracy compared to Duplex ultrasonography, utilized a thumb pulse oximeter to record plethysmographic waveforms by placing a sensor on the thumb and sequentially compressing the radial and ulnar arteries; persistent waveform loss indicated radial artery occlusion. All patients received dual antiplatelet therapy for at least 3 months, followed by long-term single antiplatelet therapy.

### Study outcomes

The procedure outcome was the rate of technical success. Technical success was defined as successful stenting with <30% residual stenosis of the target vertebral artery. Complications were categorized as major (thromboembolic events, intracranial hemorrhage, retroperitoneal hemorrhage, vessel occlusions, dissection, and perforations) or minor (puncture site hematoma or radial occlusion not requiring treatment) (16). Length of stay and post-procedure medications were extracted from the medical records. Patients were routinely interviewed by well-trained investigators with questionnaires or structured telephone interviews at 1, 3, 6, and 12 months after discharge. In-stent restenosis was defined as ≥50% stenosis of the stent or within 5 mm from the stent edge (17). Functional outcomes were assessed by modified Rankin Scale (mRS) scores, and ischemic stroke and death within 12 months after the procedure were recorded during the follow-up.

### Statistical analysis

Continuous variables were presented as mean ± standard deviation (SD) or median [interquartile range (IQR)]. Categorical variables were presented as n (%). Student’s *t*-test or Mann–Whitney U test was used to compare the differences of the continuous variables as appropriate. Furthermore, the *χ*^2^ test or Fisher’s exact test was used to compare categorical variables as appropriate. Missing values were imputed with the multiple imputation method with chain equations.

**Figure 1:**
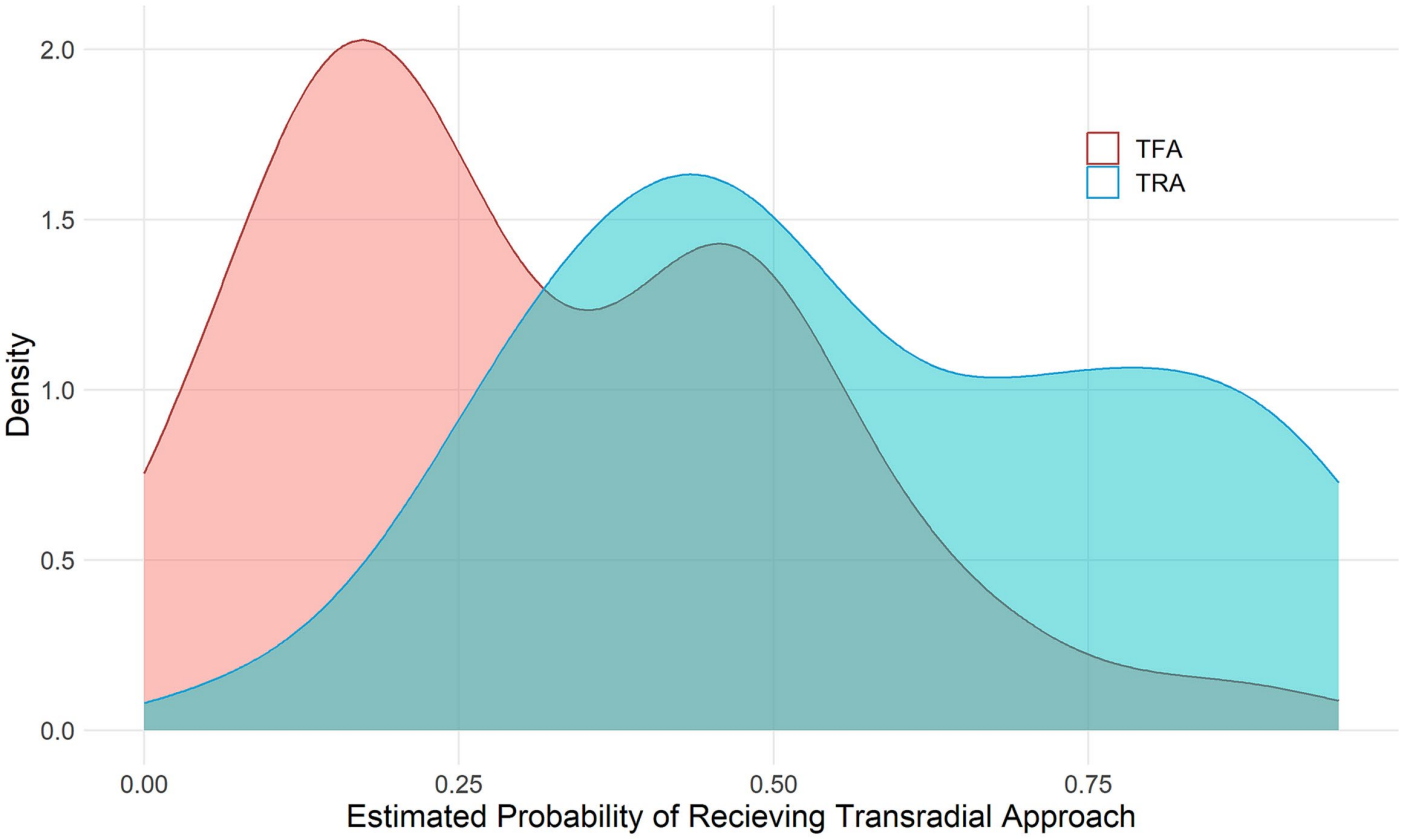
Distribution of the propensity score by the treatment approaches. TFA, transfemoral approach; TRA, transradial approach.

We compared the effects of TFA and TRA on clinical outcomes using the propensity score-matching (PSM) method with a 1:1 ratio and a caliper distance of 0.2, based on the nearest-neighbor algorithm without replacement. The propensity score was generated using all variables and listed in Table 1 through the multivariable logistic regression model. The density distribution of the estimated probability of receiving TRA is shown in Figure 1. The standardized mean difference (SMD) of the variables that generated the matched cohorts is shown in Supplementary Figure S1.

**Table 1:** Baseline characteristics of the study population according to transradial and transfemoral approaches.

Characteristics	Before matching			After matching		
	TRA (*n* = 45)	TFA (*n* = 64)	*p-*value	TRA (*n* = 32)	TFA (*n* = 32)	SMD
Age, year	65.7 (7.4)	66.5 (6.8)	0.561	66.5 (7.7)	65.9 (6.2)	0.090
Male, *n* (%)	36 (80.0)	52 (81.2)	1.000	24 (75.0)	26 (81.2)	0.152
Comorbidities, *n* (%)						
Hypertension	35 (77.8)	47 (73.4)	0.771	25 (78.1)	23 (71.9)	0.145
Diabetes mellitus	15 (33.3)	21 (32.8)	1.000	9 (28.1)	11 (34.4)	0.135
Hyperlipidemia	4 (8.9)	4 (6.2)	0.883	3 (9.4)	3 (9.4)	<0.001
Coronary heart disease	3 (6.7)	12 (18.8)	0.128	3 (9.4)	1 (3.1)	0.26
Atrial fibrillation	0 (0.0)	3 (4.7)	0.380	32 (100.0)	32 (100.0)	<0.001
Smoking	25 (55.6)	31 (48.4)	0.591	14 (43.8)	15 (46.9)	0.063
Drinking	15 (33.3)	26 (40.6)	0.567	11 (34.4)	14 (43.8)	0.193
Laboratory parameters						
Total cholesterol	3.5 [3.0, 4.7]	3.5 [3.0, 4.1]	0.399	3.5 [3.0, 4.6]	3.4 [2.9, 4.1]	0.325
Triglyceride	1.3 [1.0, 1.6]	1.2 [0.9, 1.5]	0.046	1.2 [1.0, 1.5]	1.2 [1.0, 1.5]	0.069
High-density lipoprotein	1.0 [0.9, 1.2]	1.0 [0.8, 1.1]	0.083	1.0 [0.9, 1.2]	0.9 [0.9, 1.1]	0.467
Low-density lipoprotein	1.9 [1.6, 2.8]	2.0 [1.6, 2.5]	0.682	1.9 [1.6, 2.9]	1.9 [1.6, 2.4]	0.230
Admission features						
SBP, mm Hg	135.0 [128.0, 146.0]	138.0 [128.0, 150.0]	0.68	136.5 [129.8, 146.2]	135.0 [127.8, 147.5]	0.114
DBP, mm Hg	78.8 (10.7)	80.2 (8.6)	0.436	79.0 (12.0)	79.6 (8.7)	0.057
Symptomatic, *n* (%)	25 (55.6)	43 (67.2)	0.301	20 (62.5)	18 (56.2)	0.128
NIHSS, points	0 [0, 2]	0 [0, 2]	0.323	0 [0, 2]	0 [0, 2]	0.062
mRS, points	0 [0, 1]	0 [0, 1]	0.241	0 [0, 1]	0 [0, 1]	0.130
Stent location, *n* (%)			0.378			<0.001
Left	20 (44.4)	30 (46.9)		15 (46.9)	15 (46.9)	
Right	22 (48.9)	33 (51.6)		16 (50.0)	16 (50.0)	
Bilateral	3 (6.7)	1 (1.6)		1 (3.1)	1 (3.1)	
Lesion length, mm	5.7 [4.2, 8.4]	6.1 [5.0, 7.2]	0.599	6.0 [4.7, 8.5]	6.2 [5.0, 7.3]	0.098
Lesion stenosis, %	70.0 [56.0, 78.4]	65.8 [55.9, 75.3]	0.329	70.0 [56.1, 79.1]	70.3 [54.4, 78.2]	0.089
Multiple interventions, *n* (%)			0.273			0.174
Carotid artery stenting	15 (33.3)	13 (20.3)		8 (25.0)	9 (28.1)	
Subclavian artery stenting	1 (2.2)	3 (4.7)		1 (3.1)	2 (6.2)	
Experience, *n* (%)			0.118			0.618
50–100	17 (37.8)	34 (53.1)		10 (31.2)	17 (53.1)	
100–150	17 (37.8)	23 (35.9)		12 (37.5)	12 (37.5)	
>150	11 (24.4)	7 (10.9)		10 (31.2)	3 (9.4)	
Stent type, *n* (%)			1.000			0.272
Bare metal stents	38 (84.4)	55 (85.9)		26 (81.2)	29 (90.6)	
Drug-eluting stents	7 (15.6)	9 (14.1)		6 (18.8)	3 (9.4)	
Stent technique						
Pre-dilation, *n* (%)	28 (62.2)	36 (56.2)	0.670	21 (65.6)	16 (50.0)	0.311
Pos-dilation, *n* (%)	9 (20.0)	17 (26.6)	0.573	7 (21.9)	11 (34.4)	0.404

DBP, diastolic blood pressure; mRS, modified Rankin Scale; NIHSS, National Institute of Health Stroke Scale; SBP, systolic blood pressure; SMD, standardized mean difference; TFA, transfemoral approach; TRA, transradial approach.

Due to the limited number of samples of clinical events, we used unadjusted logistic regression to calculate the odds ratio (OR) and 95% confidence interval (CI) for clinical outcomes, including complications, in-stent restenosis, and ischemic stroke or death events within 12 months after discharge. Kaplan–Meier curves were plotted for the risk of in-stent restenosis (18). In sensitivity analysis, we performed the inverse probability of treatment weighting (IPTW) method to evaluate the robustness of the logistic regression model because many patients were excluded after PSM. In addition, we used the falsification endpoint method and E-value analysis to assess the robustness of the results, which had been validated in previous reports (19, 20). Detailed information is provided in the Supplementary material. Additionally, we compared the clinical outcomes according to surgical experience and stent locations.

Statistical analysis was performed using R (version 4.2.1, R Core Team, Vienna, Austria). Two-sided *p*-values <0.05 were considered statistically significant.

## Results

During the study period, a total of 109 patients treated with endovascular treatment for the vertebral artery V1 segment stenosis were enrolled. The mean age was 66.2 ± 7 years, and 80.7% were men. A total of 45 patients were treated via TRA, and 64 patients were treated via TFA. Four cases were missing for total cholesterol, four for triglycerides, six for high-density lipoprotein, and seven for low-density lipoprotein. Little’s Missing Completely at Random (MCAR) test indicated missing at random (*p* = 0.844). The baseline characteristics of the treatment groups are listed in Table 1. They did not differ in demographics, comorbidities, admission features, surgical experience, stent types, stenting techniques, stent location, and lesion characteristics except for the level of triglycerides [TRA: 1.3 (1.0, 1.6), TFA: 1.2 (0.9, 1.5), *p* = 0.045].

**Figure 2:**
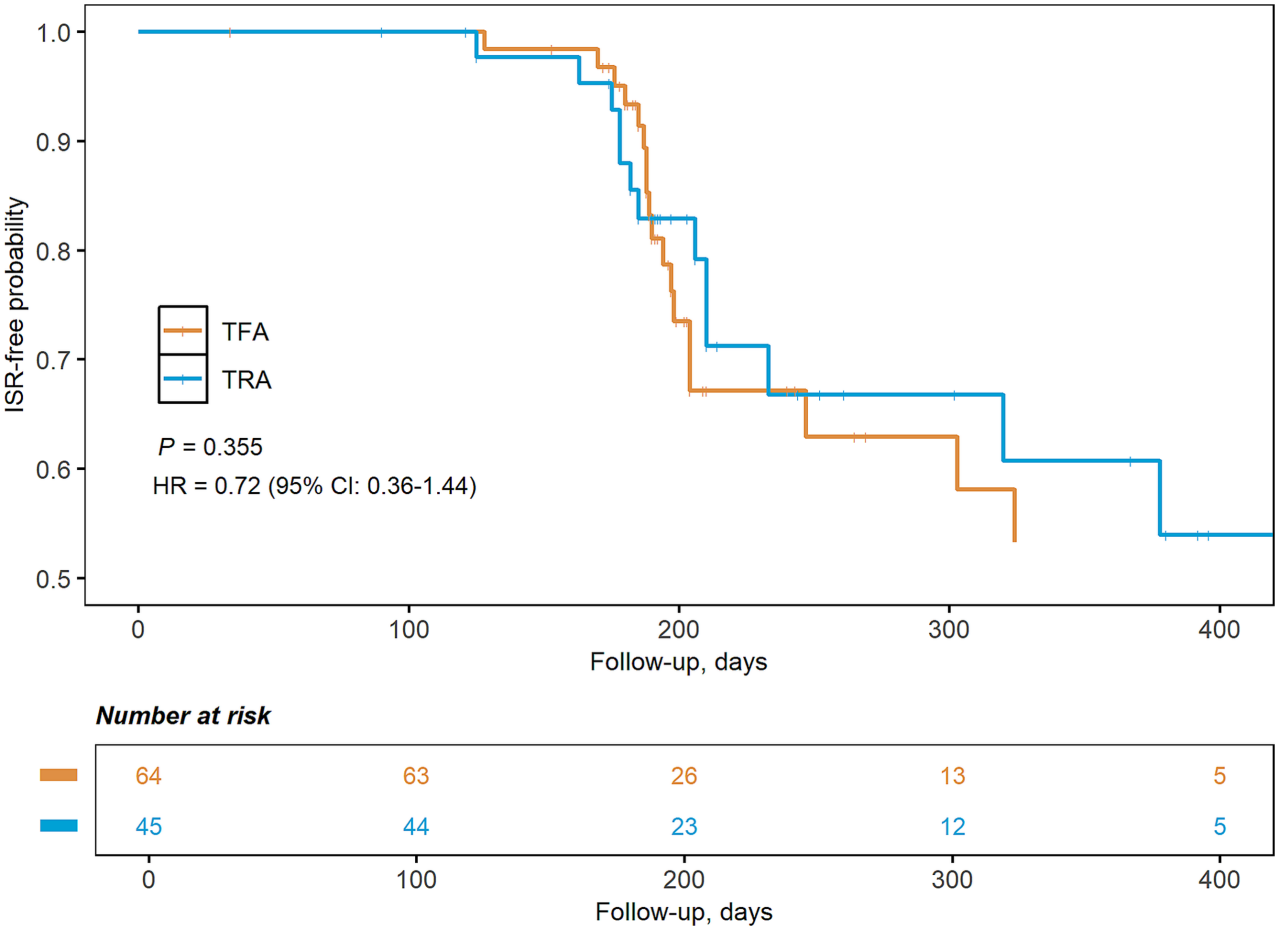
Kaplan–Meier graph of the risk of in-stent restenosis by treatment approaches. CI, confidence interval; HR, hazard ratio; TFA, transfemoral approach; TRA, transradial approach.

As listed in Table 2, the rate of technical success (TRA: 100%, TFA: 100%) and the degree of residual stenosis [TRA: 2.7% (0.9, 7.5), TFA: 3.8% (0.0, 7.2), *p* = 0.509] were similar between both treatment groups. The TRA group [43 (39, 45) min] had a significantly shorter procedure time compared to the TFA group [50 (38, 62) min, *p* = 0.005, Supplementary Figure S2]. The post-procedure complications were slightly higher in the TFA group (10.9%) than in the TRA group (2.2%), although this difference did not reach statistical significance due to the limited sample size. The rate of minor complications was 2.2% (1 radial occlusion) for the TRA group and 7.8% (5 puncture site hematoma) for the TFA group. The rate of major complications was 0.0% for the TRA group and 3.1% (1 thromboembolic event and 1 vessel dissection) for the TFA group. Post-procedure mediations were similar in both groups. The length of stay in the hospital was shorter in the TRA [9 (7, 12) days] group compared to the TFA group [10 (9, 14) days, *p* = 0.048]. Long-term outcomes were also similar between TRA [90 days mRS: 0 (0, 1) points, ischemic stroke and death: 2.2%] and TFA groups [90 days mRS: 0 (0, 1) points, ischemic stroke and death: 1.6%]. The Kaplan–Meier curve revealed a similar risk of ISR in the TRA and TFA groups (28.9% vs. 34.4%, *p* = 0.355, Figure 2).

**Table 2:** Clinical outcomes according to transradial and transfemoral approaches.

Outcomes	Before matching			After matching		
	TRA	TFA	*p-*value	TRA	TFA	SMD
Procedure outcomes						
Technical success, *n* (%)	45 (100.0)	64 (100.0)	–	32 (100.0)	32 (100.0)	<0.001
Procedure time, min	43 [39, 45]	50 [38, 62]	0.005	43 [39, 45]	50 [38, 59]	0.735
Residual stenosis, %	2.7 [0.9, 7.5]	3.8 [0.0, 7.2]	0.509	2.8 [1.2, 5.7]	4.9 [0.0, 7.5]	0.119
Post-procedure complications						
Total, *n* (%)	1 (2.2)	7 (10.9)	0.179	1 (3.1)	3 (9.4)	0.260
Minor complications, *n* (%)	1 (2.2)	5 (7.8)	0.405	1 (3.1)	3 (9.4)	0.260
Major complications, *n* (%)	0 (0.0)	2 (3.1)	0.637	0 (0)	0 (0)	<0.001
Post-procedure medication						
Antihypertensive drugs, *n* (%)	29 (64.4)	38 (59.4)	0.737	22 (68.8)	18 (56.2)	0.260
Hypoglycemic drugs, *n* (%)	15 (33.3)	18 (28.1)	0.711	9 (28.1)	10 (31.2)	0.068
Length of stay, *d*	9.0 [7.0, 12.0]	10.0 [9.0, 14.0]	0.048	9.0 [7.0, 11.0]	10.0 [7.0, 11.2]	0.342
Long-term outcomes						
90 days mRS, point	0 [0, 1]	0 [0, 1]	0.393	0 [0, 1]	0 [0, 1]	0.198
Ischemic stroke and death, *n* (%)	1 (2.2)	1 (1.6)	1.000	1 (3.1)	0 (0.0)	0.254
In-stent restenosis, *n* (%)	13 (28.9)	22 (34.4)	0.692	10 (31.2)	11 (34.4)	0.067

mRS, modified Rankin Scale; SMD, standardized mean difference; TFA, transfemoral approach; TRA, transradial approach.

After matching, the TRA group had a shorter procedure time [43 (39, 45) vs. 50 (38, 59) min, SMD = 0.735] and a shorter length of stay [9 (7, 11) vs. 10 (7, 11.2) days, SMD = 0.342]. The association between treatment groups and clinical outcomes was not significant both in the matched cohorts and IPTW analysis (post-procedure complications: OR: 0.231; 95% CI: 0.026–2.070; *p* = 0.188; ISR: OR: 0.924; 95% CI: 0.356–2.401; *p* = 0.870; ischemic stroke and death: OR: 1.909; 95% CI: 0.109–33.305; *p* = 0.655; Table 3 and Supplementary Table S1). Falsification endpoint analysis suggested that unmeasured confounders were well-balanced between treatment groups (OR: 2.079; 95% CI: 0.296–14.586; *p* = 0.458). The E-value analysis revealed that unmeasured confounders required a risk ratio of 3.528 for post-procedure complications, 1.245 for ISR, and 2.108 for ischemic stroke and death events to explain the association. Additionally, we observed that surgeons with greater surgical experience tend to favor TRA, resulting in shorter procedure times and a slightly lower incidence of surgical complications compared to those with less experience (Supplementary Table S2). Left vertebral artery stenting was associated with an increased risk of procedural complications (total: 10% vs. 5.5%; minor: 6% vs. 5.5%; major: 4% vs. 0%) compared to right vertebral artery stenting. Despite similar success rates between the groups, the mean procedure duration was marginally longer in the left stenting (45.5 min vs. 44.0 min). In the long term, the risks of ischemic stroke (4% vs. 0%) and in-stent stenosis (42% vs. 25.5%) were elevated in the left stenting. In patients treated via TRA, left vertebral artery stenting had a higher prevalence of residual stenosis, accompanied by slightly longer procedural times and a higher incidence of complications compared to right stenting (Supplementary Table S3).

**Table 3:** Treatment effects of the TRA and TFA approaches in the IPWT cohort.

Outcomes	OR (95% CI)	*p-*value	*E*-value
Post-procedure complications	0.231 (0.026, 2.070)	0.188	3.528
In-stent restenosis	0.924 (0.356–2.401)	0.870	1.245
Ischemic stroke and death	1.909 (0.109–33.305)	0.655	2.108
Falsification end point	2.079 (0.296–14.586)	0.458	2.240

CI, confidence interval; IPTW, inverse probability of treatment weighting; OR, odds ratio; TFA, transfemoral approach; TRA, transradial approach.

## Discussion

In the present study, we compared the technical feasibility and safety between TRA and TFA for vertebral artery stenting of V1 segment stenosis. We observed a similar rate of technical success and overall complications between the two approaches, as well as short procedure times and lengths of stay of TRA for stenting. To the best of our knowledge, this was the first study comparing TRA with TFA for stenting of V1 segment stenosis.

TRA is an alternative approach for endovascular procedures and has gained popularity among neurointerventionalists over the past years (10). A recent randomized controlled trial reported that TRA was associated with shorter time from sheath insertion to stent insertion and higher patient acceptance and satisfaction (21). Schartz et al. conducted a meta-analysis to investigate the difference in complication rates between TRA and TFA for neurovascular procedures. After analyzing 17 studies, they reported that the rates of access site complications were 1.8 and 3.2% for TRA and TFA, respectively (22). Catapano et al. performed a retrospective analysis among 877 patients receiving neuroangiographic procedures and reported that the overall complication rate with TFA procedures [60/844 (7%)] was significantly higher than TRA procedures [4/206 (2%)] (16). In our study, we reported that TFA procedures had a slightly higher complication rate compared to TRA procedures, although this difference did not reach statistical significance due to the small sample size. Our results also indicated that the length of stay was significantly shorter in TRA procedures. Literature in cardiology suggested that TRA was associated with a reduction in the post-procedural length of stay for acute myocardial infarction patients undergoing rescue angioplasty (TRA: 7.0, TFA: 7.9 days, *p* < 0.05) (23). Ge and Wei (24) reviewed 1,048 cerebral angiograms and found that the duration of hospital stay was shorter in the TRA group (123.8 h) compared to the TFA group (168.7 h, *p* < 0.05).

Endovascular treatment for V1 segment stenosis of the vertebral artery is generally preferred over aggressive medical management due to the specific location of the segment, which is challenging to access surgically (25, 26). Recently, the Vertebral Artery Ischemic Stenting Trial (VIST) revealed that stenting for the extracranial vertebral artery was safe with low complications and might have potential efficacy for preventing stroke recurrence (27). However, the main problem with stent therapy is the high incidence of ISR, which varies from 15 to 60% according to different study designs (8, 28). Our results found that TRA did not increase the incidence of ISR after stenting for the V1 segment (28.9% vs. 34.4%). Che et al. (2) investigated the short- and long-term outcomes after stenting for V1 segment stenosis and revealed that the treatment approach was not included in the final multivariable analysis. Guo et al. (29) conducted a retrospective analysis of a multicenter registry among patients with symptomatic intracranial vertebrobasilar artery stenosis treated with TRA or TFA, reporting an incidence of symptomatic ISR of 6% for the TRA group and 13.1% for the TFA group (*p* = 0.692). These results supported the potential safety and efficacy of TRA for the treatment of vertebrobasilar artery stenosis. Notably, anatomic factors may influence the outcome of vertebral artery stenting. A previous review reported that placement of stents on the left vertebral artery via TRA is affected by the angle and distance between the innominate and left subclavian arteries (30). In our study, left-sided procedures were associated with a higher rate of procedural complications despite similar technical success rates for both left and right approaches. The mean procedure duration was slightly longer for left-sided stenting. In addition, the left vertebral artery is associated with increased risks of ischemic stroke and in-stent stenosis, and patients treated via TRA showed a higher prevalence of residual stenosis.

Despite the technical safety and patient preference for TRA, TFA remains the preferred choice among interventional neurologists (31). Factors limiting the promotion of TRA may include the steep learning curve and the lack of evidence from large randomized controlled trials. Wilkinson et al. retrospectively examined 500 cerebral angiograms and recorded the fluoroscopy time at different stages of surgery proficiency. They suggested 30–50 cases of angiograms would be needed to pass the steepest stage of the learning curve (11). Zussman et al. (32) evaluated the safety and feasibility of TRA in 50 diagnostic cerebral arteriograms and found that neurointerventionalists tend to achieve higher success rates after performing 50 cases. Almallouhi et al. (33) found that the number of crossovers from TRA to TFA rapidly declined after 6 weeks of training. In the present study, procedures were performed by seasoned neurointerventionalists who had navigated beyond the inflection point of the learning curve. The procedure time was notably shorter for the TRA group than for the TFA group, with more experienced neurointerventionalists demonstrating reduced procedure times.

This study had several limitations. First, this was a retrospective study with a small sample size, and the selection of TRA or TFA was based on experience and preference, which may have generated selection biases. Second, this study lacks standard protocols for screening puncture site complications and may underestimate the incidence of radial occlusion, which requires Doppler ultrasound examination. Third, we were unable to evaluate the learning curve of neurointerventionalists participating in this study because of the heterogeneity of each center. Fourth, due to the retrospective nature of the study, we were unable to provide information about patient-reported outcomes or satisfaction scores, which might reflect the additional advantages of TRA. Finally, our study’s sample size was relatively small, particularly when assessing outcomes that were rare but clinically significant, which may have led to limited statistical power and a Type II error.

In conclusion, our study found that TRA was a safe and feasible approach for vertebral artery stenting of V1 segment stenosis. TRA had an advantage over TFA in terms of reduced procedure time and length of stay. Further large-scale and randomized controlled studies were warranted to verify the findings, given the retrospective and non-randomized nature of this study.

## Data Availability

The raw data supporting the conclusions of this article will be made available by the authors, without undue reservation.
