# Supplementary material for: Transradial approach in vertebral artery stenting
Source: Front Neurol. 2025 Sep 12;16:1643105. doi: 10.3389/fneur.2025.1643105 (PMC12463936; doi:10.3389/fneur.2025.1643105)
Supplement: Supplementary file 1 [file Data_Sheet_1.PDF]

Supplementary materials

Supplementary methods

Unmeasured bias analysis

To evaluate the robustness of the results, we performed falsification endpoint method and E value analysis that were reported in previous studies[1]. In the falsification endpoint method, we would select an endpoint (composite of pneumonia and urinary tract and gastrointestinal infection at angiographic follow-up) that was not causally related to the intervention treatment (TFA vs TRA), and performed similar statistical analyses to other outcomes. When we observed no treatment effect for the falsification endpoint, it would support that there might be a casual treatment effect for the outcomes. E-value analysis would identify the minimum strength required for unmeasured confounders to affect treatment and clinical outcomes and to fully explain the relationship based on measured covariates, which means that the impact of unmeasured confounder on the relationship between treatment and clinical outcomes would be tolerable when the calculated relative risk value is bigger than previous reports[2].

Figure 1. Balance of covariates between TRA and TFA groups.

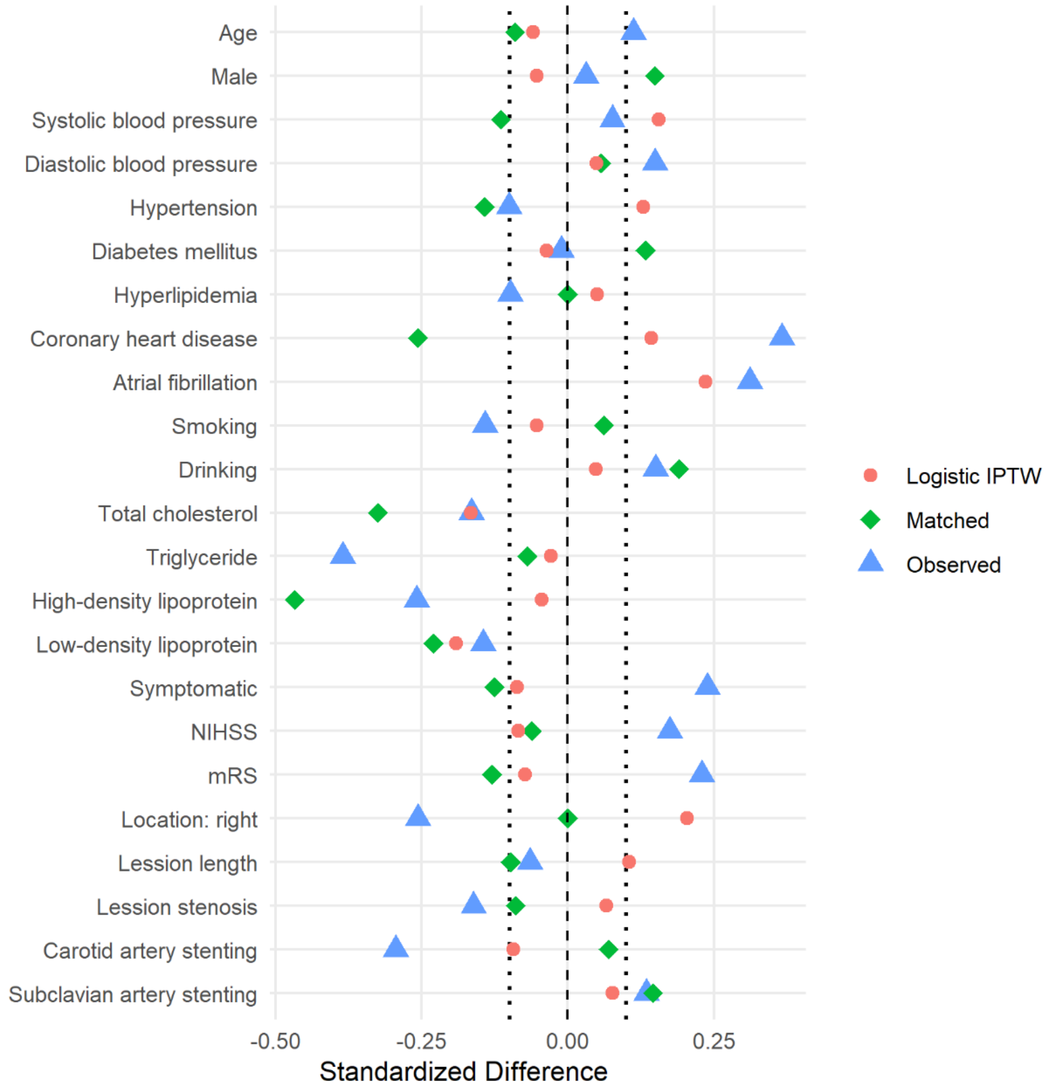

Figure 2. Difference of the procedure time between TRA and TFA before and after PSM.

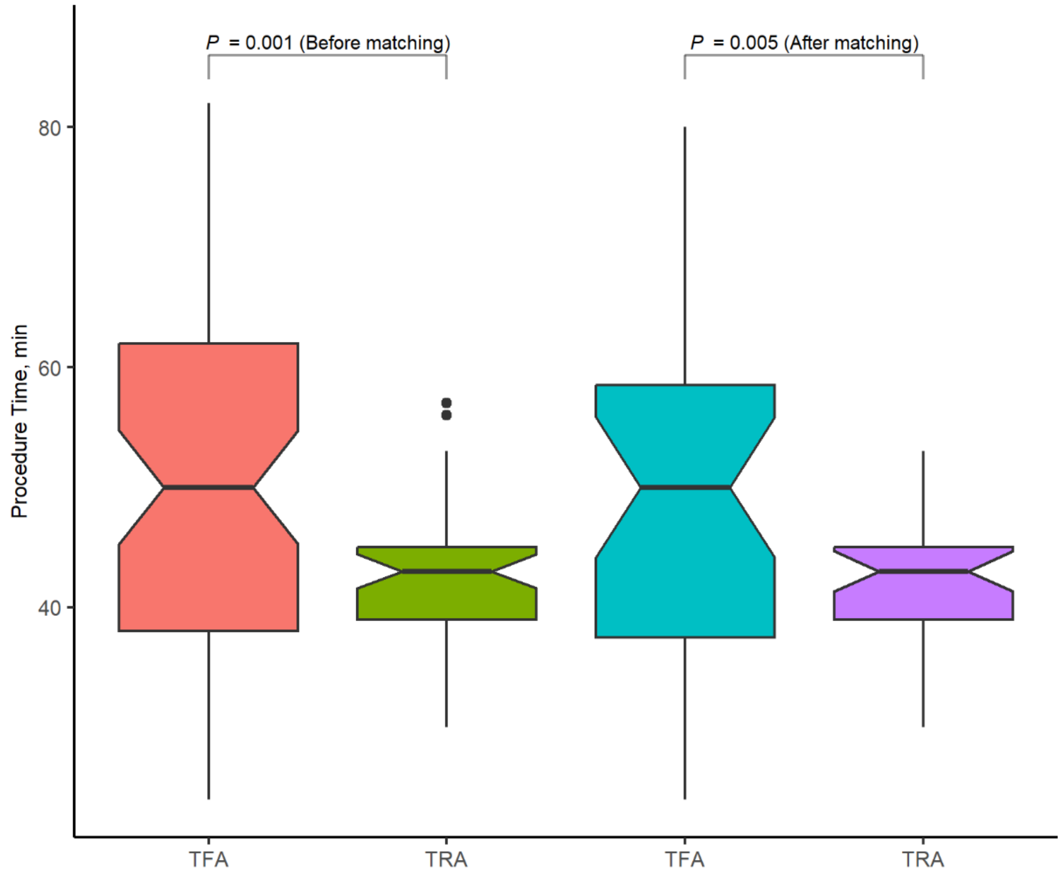

Abbreviations: PSM, propensity score matching;TFA, transfemoral approach; TRA, transradial approach. P value was calculated based on the Mann-Whitney U test.

Table 1. Treatment effects of the TRA and TFA in the PSM cohort.

Outcomes	OR (95% CI)	*P* value	E value
Complications	0.312 (0.015-2.592)	0.325	2.980
In-stent restenosis	0.868 (0.302-2.475)	0.790	1.245
Ischemic stroke and death	-	-	-
Falsification end point	3.207 (0.386-66.859)	0.325	2.981

Abbreviations: CI, confidence interval; OR, odds ratio; PSM, propensity score matching; TFA, transfemoral approach; TRA, transradial approach.

Table 2. Clinical outcomes according to surgical experience.

**Outcomes**	**50-100**	**100-150**	**>150**	***P* value**
Procedure outcomes				
Technical success, n (%)	51 (100.0)	40 (100.0)	18 (100.0)	1.000
Procedure time, min	48.0 [42.0, 62.0]	42.5 [35.5, 50.0]	43.0 [34.5, 45.8]	0.001
Residual stenosis, %	1.1 [0.0, 6.1]	4.3 [0.7, 8.3]	3.3 [1.2, 9.4]	0.245
Post-procedure complications				
Total, n (%)	4 (7.8)	4 (10.0)	0 (0.0)	0.394
Minor complications, n (%)	3 (5.9)	3 (7.5)	0 (0.0)	0.504
Major complications, n (%)	1 (2.0)	1 (2.5)	0 (0.0)	0.803
Post-procedure medication				
Antihypertensive drugs, n (%)	33 (64.7)	24 (60.0)	10 (55.6)	0.768
Hypoglycemic drugs, n (%)	13 (25.5)	12 (30.0)	8 (44.4)	0.322
Length of stay, d	10.0 [8.0, 12.0]	10.0 [7.0, 15.0]	10.0 [8.0, 14.0]	0.980
Long-term outcomes				
90 days mRS, point	0.0 [0.0, 1.0]	0.0 [0.0, 1.0]	0.5 [0.0, 1.0]	0.113
Ischemic stroke and death, n (%)	2 (3.9)	0 (0.0)	0 (0.0)	0.314
In-stent restenosis, n (%)	19 (37.3)	10 (25.0)	6 (33.3)	0.459

Abbreviations: mRS, modified Rankin Scale.

Table 3. Clinical outcomes according to stent location.

Total population	Left	Right	Bilateral	*P* value
Procedure outcomes				
Technical success, n (%)	50 (100.0)	55 (100.0)	4 (100.0)	1.000
Procedure time, min	45.5 [40.0, 54.0]	44.0 [38.5, 51.0]	44.5 [42.8, 50.8]	0.871
Residual stenosis, %	2.9 [0.0, 8.6]	2.9 [0.0, 6.9]	1.2 [0.7, 2.8]	0.784
Post-procedure complications				
Total, n (%)	5 (10.0)	3 (5.5)	0 (0.0)	0.570
Minor complications, n (%)	3 (6.0)	3 (5.5)	0 (0.0)	0.879
Major complications, n (%)	2 (4.0)	0 (0.0)	0 (0.0)	0.301
Post-procedure medication				
Antihypertensive drugs, n (%)	28 (56.0)	36 (65.5)	3 (75.0)	0.520
Hypoglycemic drugs, n (%)	15 (30.0)	17 (30.9)	1 (25.0)	0.968
Length of stay, d	10.0 [7.0, 12.0]	10.0 [8.0, 14.5]	10.0 [8.5, 11.8]	0.610
Long-term outcomes				
90 days mRS, point	0.0 [0.0, 1.0]	0.0 [0.0, 1.0]	0.0 [0.0, 0.2]	0.681
Ischemic stroke and death, n (%)	2 (4.0)	0 (0.0)	0 (0.0)	0.301
In-stent restenosis, n (%)	21 (42.0)	14 (25.5)	0 (0.0)	0.072
**TRA**	**Left**	**Right**	**Bilateral**	***P* value**
Procedure outcomes				
Technical success, n (%)	20 (100.0)	22 (100.0)	3 (100.0)	1.000
Procedure time, min	43.5 [39.5, 46.0]	42.5 [39.2, 44.8]	44.0 [41.5, 44.5]	0.877
Residual stenosis, %	5.7 [1.9, 11.8]	2.6 [0.0, 4.0]	0.9 [0.4, 1.2]	0.039
Post-procedure complications				
Total, n (%)	1 (5.0)	0 (0.0)	0 (0.0)	0.528
Minor complications, n (%)	1 (5.0)	0 (0.0)	0 (0.0)	0.528
Major complications, n (%)	0 (0.0)	0 (0.0)	0 (0.0)	1.000
Post-procedure medication				
Antihypertensive drugs, n (%)	13 (65.0)	14 (63.6)	2 (66.7)	0.992
Hypoglycemic drugs, n (%)	6 (30.0)	8 (36.4)	1 (33.3)	0.909
Length of stay, d	9.0 [7.0, 12.5]	8.5 [7.2, 10.8]	9.0 [8.0, 11.5]	0.914
Long-term outcomes				
90 days mRS, point	0.0 [0.0, 1.0]	0.0 [0.0, 1.0]	0.0 [0.0, 0.5]	0.858
Ischemic stroke and death, n (%)	1 (5.0)	0 (0.0)	0 (0.0)	0.528
In-stent restenosis, n (%)	7 (35.0)	6 (27.3)	0 (0.0)	0.447

Abbreviations: mRS, modified Rankin Scale.

Reference

1. Majmundar M, Kumar A, Doshi R, Shariff M, Krishnaswamy A, Reed GW, Brockett J, Lahorra JA, Svensson LG, Puri R, et al. Early outcomes of transcatheter versus surgical aortic valve implantation in patients with bicuspid aortic valve stenosis. *EuroIntervention*. 2022;18:23–32.

2. Haneuse S, VanderWeele TJ, Arterburn D. Using the E-Value to Assess the Potential Effect of Unmeasured Confounding in Observational Studies. *JAMA*. 2019;321:602–603.
